# Histochemical Investigation and Kinds of Alkaloids in Leaves of Different Developmental Stages in *Thymus quinquecostatus*

**DOI:** 10.1155/2014/839548

**Published:** 2014-07-01

**Authors:** Haiting Jing, Jing Liu, Hanzhu Liu, Hua Xin

**Affiliations:** ^1^College of Life Sciences, Qingdao Agricultural University, Qingdao 266109, China; ^2^Central Laboratory, Qingdao Agricultural University, Qingdao 266109, China; ^3^University Key Laboratory of Plant Biotechnology, Shandong, Qingdao 266109, China

## Abstract

*Thymus quinquecostatus*, with more medical value, is a kind of wild plants. In order to exploit and utilize this plant, we studied the species and locations of alkaloids in its leaves. In this paper, histochemical study of leaves at different developing stages was taken to localize the alkaloids. Meanwhile, the kinds and content of alkaloids in leaves were identified using GC-MS technique. It was found that there were two kinds of glandular trichomes, namely, peltate trichomes and capitate trichomes, on the surface of leaves, and their secretory cells could secrete alkaloids. Results showed that trichomes could secrete alkaloids as soon as the first pair of leaves formed, and there were altogether 18 kinds of alkaloids identified by GC-MS. Nearly all of these alkaloids of leaves at different developing stages were distinct from each other, except one, 3-methoxy-a-methyl-benzeneethanamine, persists at different developing stages with high concentration.

## 1. Introduction

Plants may synthesize many secondary metabolites, which can help plants survive and reproduce in the natural environment [1]. Alkaloids are a group of important secondary metabolites. They are relatively simple molecules and are present in plants at <10 g/kg [2]. Many alkaloids are toxic, which are used for plants to protect themselves against the aggression from other organisms, and this instinct action is an important ecological function. Alkaloids can be produced by a large variety of organisms, especially most abundant in higher plants. At least 25% of higher plants contain these metabolites [3]. Many kinds of alkaloids are effective ingredients in most medicinal plants, so that they are applied in the treatment of diseases in traditional herbal medicine. Alkaloids, with obviously physiological activity, have lots of functions, such as anesthesia, analgesia, antibacterial and antiviral effects, antineoplastic activity, antihypertensive effect, insecticidal action, and so forth. They have already been applied in widespread use in the medical and agricultural industries [4–8]. So alkaloids have been and continue to be the object of human interest concerning new possibilities for their safe utilization and ensuing health benefits [3].

Some plant families, such as Apocynaceae, Papaveraceae, and Ranunculaceae are especially rich in alkaloids [9]. The Lamiaceae, a big family, contains more than 3500 kinds of plants, and there are many species, such as* Mentha haplocalyx*,* Perilla frutescent, *and* Lavandula angustifolia,* notable for their essential oil in this family [10, 11]. Except essential oil, plants in this family can also produce alkaloids, for example, leonurine and stachydrine, with many physiological functions [12, 13].* Thymus quinquecostatus*, commonly called thyme, is a scrubby subshrub of* Thymus* in Lamiaceae, and this kind of plant has ornamental and medical value, such as anti-inflammatory and analgesic properties. In some regions of China, people often burn* Thymus quinquecostatus* to keep off mosquitoes. There are many glandular trichomes on the leaf surface, and they can secrete essential oil, phenols, and flavone [14]. When we studied the development and excretion of trichomes, we found that the leaves could also secrete alkaloids. The purpose of the present work was to have a preliminary study on the alkaloids of leaves at different developing stages.

## 2. Materials and Methods

### 2.1. Materials

*Thymus quinquecostatus* were collected from Laoshan Mountain of China in September 2013, and then they were cultured in the plant laboratory of Qingdao Agricultural University. The oval leaves of them are opposite. The fresh young leaves (the first couple and the second one from the top), nearly mature leaves (the third couple from the top), and the mature leaves (the forth couple from the top) were, respectively, picked. The length and width of blades were measured with a vernier caliper. In the experiment, three replicates of leaves from three plants were measured.

### 2.2. Histochemical Identification of Alkaloids

#### 2.2.1. Blade Staining Method

Whole fresh leaves at different developing stages were separately put on a glass slide and dyed with a few drops of improving Dragendorff reagent for 2–4 minutes. Then they were rinsed with distilled water. After covered with a coverslip, leaves were observed with an OLYMPUS BH-2 microscope. Undyed mature leaves were as control.

#### 2.2.2. Frozen Section Staining Method

Fresh leaves at different developing stages were separately embedded in agar (4%) and sectioned (20 *μ*m) with a MICROM HM525 freezing microtome. The leaf sections were covered with a few drops of improving Dragendorff reagent for 20–30 seconds. Then they were rinsed with distilled water. After covered with a coverslip, the sections were observed with an OLYMPUS BH-2 microscope. The undyed section of a mature leaf was as control.

### 2.3. Alkaloids Analysis

0.9 g leaves at different developing stages was picked and naturally dried. The dried leaves were extracted with CH_2_Cl_2_ for 4 times, and at each time there were ultrasonic extraction for 30 minutes and solvent extraction for an hour and a half. The ultrasonic conditions were 30 KHz, 150 W, and 25°C. The extracts were mixed four times and concentrated by a rotary evaporator. The volume was metered to 5 mL with CH_2_Cl_2_. The alkaloids were qualitatively and quantitatively analyzed with gas chromatography-mass spectrometry (GC-MS). The GC-MS (Agilent 7890A/5975C, Swindon, UK) was equipped with an DB-5 capillary column (50 × 0.32 mm ID × 0.25 *μ*m film thickness) and the data were taken under the following conditions: initial temperature 40°C, temperature ramp 15°C per min, to 300°C (holding for 5 min). The autosampler transferred the liquid sample directly to the column. The carrier gas was helium and the velocity was 1.0 mL per min. The main components of the qualitative and quantitative results were identified through the mass spectrometry database (NIST08). For confirmation of results, the extract was analyzed for three replications.

## 3. Result

### 3.1. Size of Different Leaves

For size of different leaves, see Table 1.

### 3.2. Histochemical Localization of Alkaloids in Leaves of* Thymus quinquecostatus*

**Figure 1:**
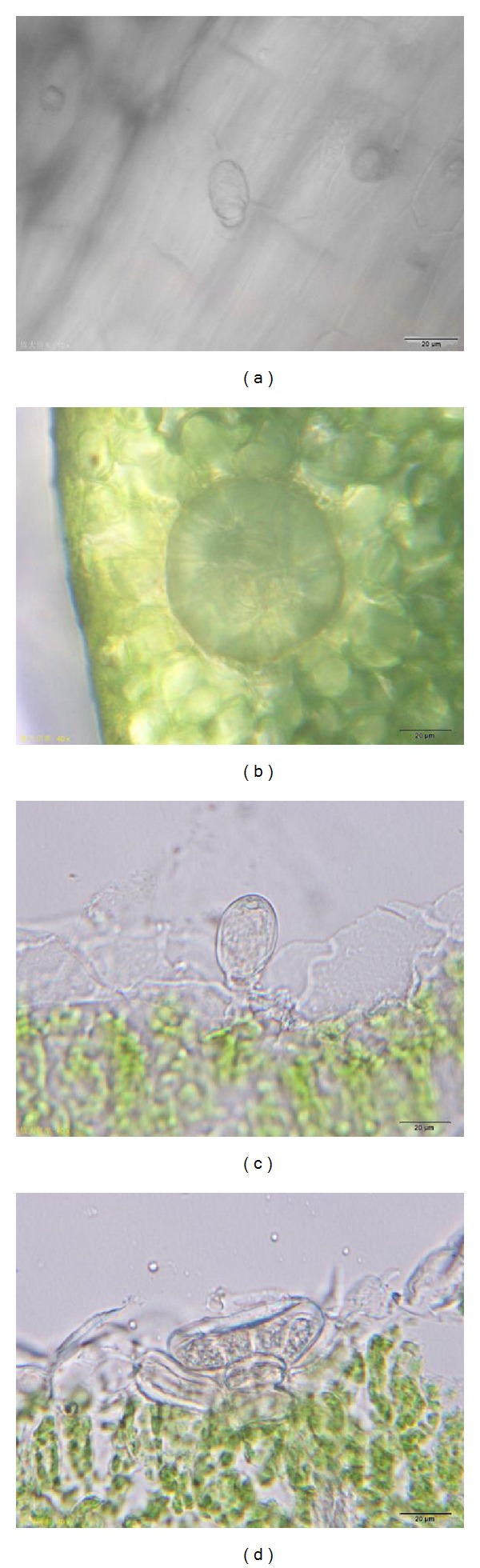
(a)-(b) Surface view of a mature leaf of* T. quinquecostatus*. (a) A capitate trichome. (b) A peltate trichome. (c)-(d) Transverse sections of a mature leaf of* T. quinquecostatus*. (c) Structure of a capitate trichome structure. (d) Structure of a peltate trichome.

Under the microscope, two kinds of glandular trichomes on blades could be seen on surface view. They were capitate trichomes and the peltate trichomes (Figures 1(a) and 1(b)). The capitate trichome, which consisted of a basal cell, a stalk cell, and a unicellular head, was small (Figure 1(c)). The width of its head was about 16.81 *μ*m. The peltate trichome, which consisted of a basal cell, a stalk cell, and a multicellular head was large (Figure 1(d)). The width of its head containing 12 secretory cells was about 61.20 *μ*m.

**Figure 2:**
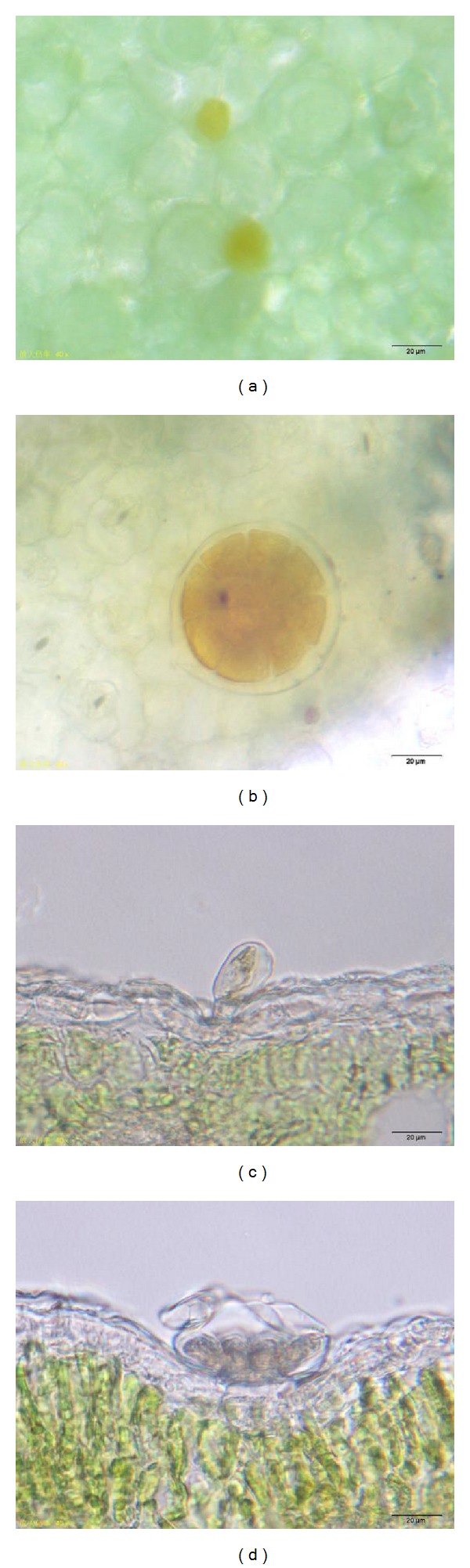
(a)-(b) Surface view of a mature leaf of* T. quinquecostatus*. (a) A capitate trichome dyed yellow. (b) A peltate trichome dyed yellow. (c)-(d) Transverse sections of a mature leaf of* T. quinquecostatus.* (c) The secretory cell of a capitate trichome dyed yellow. (d) The secretory cells of a peltate trichome dyed yellow.

Both capitate trichomes (Figure 2(a)) and peltate trichomes (Figure 2(b)) were orange after dyed with Dragendorff reagent. The epidermis of leaves was colourless. From transverse section view of a mature leaf, we could see that the secretory cell of a capitate trichome was covered with intact cuticle. The interior of a secretory cell showed yellow after dyed with Dragendorff reagent, but the basal cell and the talk cell were nearly colourless (Figure 2(c)). The head of a peltate trichome was also covered with cuticle, yet the cuticle had cracked. The interior of 12 secretory cells showed yellow after dyed with Dragendorff reagent, the basal cell and the talk cell were nearly colourless (Figure 2(d)). This was the same with the capitate trichome. Except secretory cells of glandular trichomes, other parts, such as mesophyll cells and leaf veins, were not dyed yellow.

**Figure 3:**
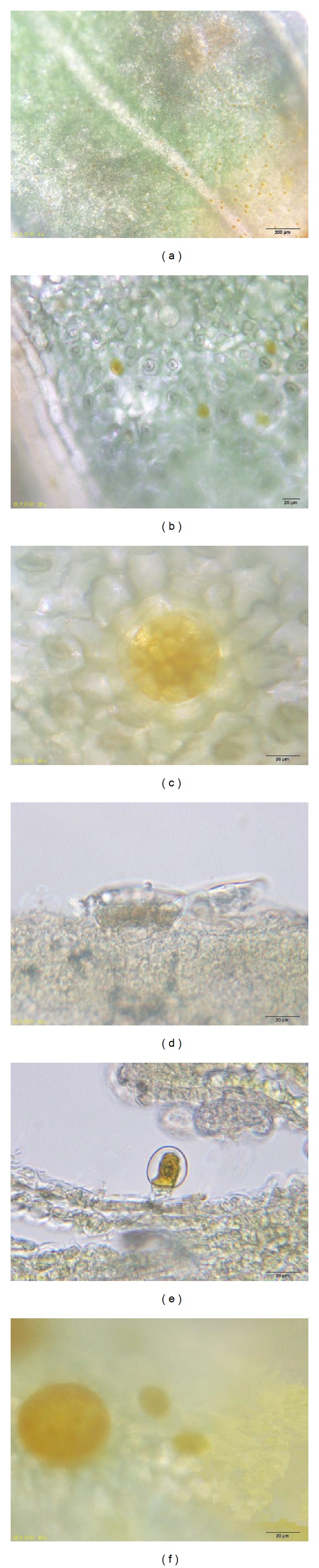
(a)-(b) Surface view of young leaves of* T. quinquecostatus*. (a) A lot of capitate trichomes dyed yellow. (b) Peltate trichomes showed colourless after dyed. (c) A peltate trichome showed yellow after dyed. (d) Transverse section of a young leaf of* T. quinquecostatus, *showing the head of a peltate trichome dyed yellow. (e) Transverse section of a young leaf of* T. quinquecostatus, *showing the head of a capitate trichome dyed yellow. (f) Surface view of a nearly mature leaf of* T. quinquecostatus*, showing capitates trichomes and the peltate trichomes dyed yellow.

Leaves at different developing stages were dyed with Dragendorff reagent. The alkaloids were produced at an early stage. They could be seen in trichomes of the first couple of young leaves. The capitate trichomes were easier dyed than the peltate trichomes (Figures 3(a) and 3(b)). As the dying time went on, the peltate trichomes showed yellow too, but the color was shallow (Figures 3(c) and 3(d)). The transverse sections of young leaves showed that the secretory cells of both the capitates trichomes and the peltate trichomes were yellow. As the leaves gradually grew to be mature, the coloring time of capitate trichomes was very closed to that of peltate trichomes (Figures 3(e) and 3(f)).

### 3.3. Alkaloids in Leaves

Leaves at different developing stages of* Thymus quinquecostatus* had different kinds of alkaloids (Table 2). In Total eighteen kinds of alkaloids were identified in leaves. Young leaves had nine kinds of alkaloids and the content of 3-phenyl-piperidine was the highest, 11.49%. Nearly mature leaves had six kinds of alkaloids and the content of 3,7-Dimethyl-1,6-octadien-3-yl 2-aminobenzoate was the highest, 3.70%. Mature leaves had eight kinds of alkaloids and the content of 3-methoxy-a-methyl-benzeneethanamine was the highest, 3.52%, and this kind of alkaloids could be commonly found in leaves at different developing stages. There were seven kinds of alkaloids in the young leaves, three kinds of alkaloids in the nearly mature leaves, and four kinds of alkaloids in the mature leaves that were unique to alkaloids in other developing stages of leaves, respectively. It can be implied that with the development of leaves, great changes had taken place in the kinds of alkaloids in glandular trichomes.

## 4. Discussion

Alkaloids can be found in many plant organs, such as roots, stems, leaves, fruits, and seeds. Dhale studied alkaloids in three species:* Adhatoda zeylanica*,* Ruta graveolens, *and* Vitex negundo. *It was shown that alkaloids existed in the epidermis, mesophyll cells, and parenchyma cells of the leaf veins [15]. The histochemical studies of leaves of* Mimusops elengi *and* Syzygium cumini *showed that alkaloids could be observed in the mesophyll cells of leaves of two species [16]. There are a lot of glandular trichomes on the surface of leaves in many species of plants. Glandular trichomes are renowned as prolific “chemical factories” for either synthesizing or storing plant metabolites as chemical defenses [17], and alkaloids are one kind of important materials in them. Abiola had extracted a new kind of alkaloid from glandular trichomes on leaves of* Mucuna pruriens *[18]. Nicotine mostly existed in the trichomes of* Nicotiana*, such as* Nicotiana tabacum* and* Nicotiana stocktonii *[19, 20]. From these researches, we could see that alkaloids of leaves are mainly distributed in epidermis (protective tissue), parenchyma, and secretory structures. In our studies, the alkaloids of leaves of* Thymus quinquecostatus* could be observed in the glandular trichomes. Both peltate trichomes and capitate ones secreted alkaloids. At the early developing stage of leaves, alkaloids could be found in the young trichomes. We did not find insects or diseases either in wild plants or in cultivated plants in laboratory during the investigation process. It is still not clear whether this phenomenon is related to the alkaloids of* Thymus quinquecostatus,* so the method of pathology is valuable for further study. In addition, the difference of alkaloids between two kinds of glandular trichomes on leaves of* Thymus quinquecostatus* also needs further study.

Alkaloids have a wide distribution in the nature, and nearly 10 thousands of alkaloids have been found. They can be classified as organic amine alkaloids, piperidine alkaloids, indolizidine alkaloids, quinolizidine alkaloids, pyrrolizidine alkaloids, izidine alkaloids, pyrrolidine alkaloids, tropane alkaloids, indole alkaloids, and so on [3]. The distribution of alkaloids has specificity in plant taxa. There may be large differences among alkaloids in the plants of one family, but alkaloids may be similar in the species of the same genus. For example, reserpine, one kind of indole alkaloids, which has antihypertensive effect, is produced by the plants of the genus* Rauvolfia *(Apocynaceae) [21]. Morphine, an isoquinoline alkaloid, which has analgesic and anesthetic effect, is produced by the plants of the genus* Papaver* (Papaveraceae) [22]. Cephalotaxine, which has remarkable anticancer effect, is produced by plants of the genus* Cephalotaxus* (Cephalotaxaceae) [23]. But for the moment, little research has been done on the alkaloids of plants in the Labiatae. Research showed that leonurine, which is an organic amine alkaloid and has been widely used, is mainly produced by plants in the genus* Leonurus* [24]. The plants of Labiatae can also produce other kinds of alkaloids, like hederacine A and hederacine B [25], phenolic alkaloids [26], diterpenoid alkaloids [27], and so on. In this paper, the results indicated that nearly all kinds of the nine alkaloids in young leaves of* Thymus quinquecostatus* were organic amine alkaloids, except one, 3-phenyl-piperidine, belonged to piperidine alkaloids, and all of the six kinds of alkaloids in nearly mature leaves and seven kinds of alkaloids in mature leaves belonged to organic amine alkaloids. So organic amine alkaloids are dominant in the alkaloids of* Thymus quinquecostatus*, but whether organic amine alkaloids are the main type of alkaloids produced by plants of Labiatae requires further study.

## Figures and Tables

**Table 1:** The size of leaves at different developing stages of *T.  quinquecostatus*.

Leaves	Length (mm)	Width (mm)
The first couple of young leaves	0.81 ± 0.08	0.78 ± 0.08
The second couple of young leaves	2.32 ± 0.16	1.25 ± 0.03
Nearly mature leaves	5.92 ± 0.05	2.80 ± 0.01
Mature leaves	7.87 ± 0.19	3.95 ± 0.15

**Table 2:** Content of alkaloids in different developing stages of leaves of *Thymus quinquecostatus*.

Alkaloids	Young leaf	Nearly mature leaves	Mature leaves
P-methylamphetamine	3.11 ± 0.05	/	/
L-(-)-ephedrine	3.39 ± 0.15	/	/
Methanimidamide,N,N-dimethyl -N′-phenyl-	4.38 ± 0.03	/	/
Piperidine, 3-phenyl-	11.49 ± 0.07	/	/
2-Heptanol,6-amino-2-methyl-	3.49 ± 0.12	/	/
1-Hexanamine, N-methyl-	3.19 ± 0.04	/	/
Benzenemethanol, a-(1-aminoethyl) -4-hydroxy-	2.53 ± 0.15	/	/
3-methoxy-a-methyl-benzeneethanamine	5.08 ± 0.03	2.24 ± 0.07	3.52 ± 0.10
1-Octadecanamine,N-methyl-	7.21 ± 0.06	/	0.10 ± 0.03
3,7-Dimethyl-1,6-octadien-3-yl	/	3.70 ± 0.10	/
2-aminobenzoate			
Methoxyamphetamine	/	0.37 ± 0.07	/
3-Azabicyclo[3.2.2]nonane	/	1.00 ± 0.05	/
Benzeneethanamine,2,3-dimethoxy -a-methyl-	/	0.93 ± 0.04	1.68 ± 0.07
2-Nonadecanamine	/	1.61 ± 0.11	1.89 ± 0.11
Benzeneethanamine a-methyl-	/	/	1.39 ± 0.03
Norpseudoephedrine	/	/	1.53 ± 0.08
4-Ethoxyamphetamine	/	/	2.49 ± 0.04
1-Propanamine,3-(10,11-dihydro -5H-dibenzo[a,d]cyclohepten-5-ylidene)-N-methyl-	/	/	1.19 ± 0.02
